# Feasibility, acceptability, and effectiveness of young people-specific, integrated out-of-hospital services: a protocol for a systematic review

**DOI:** 10.1186/s13643-019-0993-9

**Published:** 2019-03-28

**Authors:** Anuja Pandey, Aswathikutty Gireesh, Russell Viner

**Affiliations:** UCL Great Ormond Street Institute of Child Health, Population, Policy and Practice Programme, 30 Guilford Street, London, WC1N 1EH UK

**Keywords:** Integrated health services, Young people, Out-of-hospital, Health service delivery

## Abstract

**Background:**

The need for specific services for young people is being widely recognized to address their unique and complex health needs. Growing evidence in integrated health services shows promise in improving the efficiency of health systems. Although there is a broad agreement on the need for integrated care in young people, there has been no systematic effort to evaluate the provision of integrated out-of-hospital health services for this group. The proposed systematic review aims to assess the effectiveness, feasibility, and acceptability of young people-specific integrated out-of-hospital services.

**Methods:**

We will search the following databases using a systematic search strategy: MEDLINE, EMBASE, CINAHL Plus, and CENTRAL for articles published in the English language without applying date filters. The search will be supplemented with article search from systematic reviews of relevant topics, reference lists, and citations of included studies. Eligible studies will include peer-reviewed publications reporting on the evaluation of integrated out-of-hospital health services for young people (10–24 years) regarding effectiveness, feasibility, and acceptability. Two reviewers (AP and AA) will independently carry out study selection, data extraction, and quality assessment. Study findings will be summarized in a narrative review. Wherever possible, evidence synthesis of quantitative data will be done using forest plots and pooled estimates.

**Discussion:**

This review aims to provide comprehensive evidence regarding young people-specific integrated out-of-hospital health services. Such rigorously evaluated evidence will be useful for policy makers and health professionals to design and select health services for this group. This review will also identify any evidence gaps in young people-specific integrated health services evaluation.

**Systematic review registration:**

PROSPERO CRD42017068836

**Electronic supplementary material:**

The online version of this article (10.1186/s13643-019-0993-9) contains supplementary material, which is available to authorized users.

## Background

There are 1.8 billion young people in the world, more than ever before, providing an unprecedented opportunity for social and economic progress [1]. Estimates suggest that approximately 25% of the world’s population is between 10 and 24 years of age [2]. A report, based on the WHO’s 2004 Global Burden of Disease (GBD) study, recorded 236 million incident DALYs (disability-adjusted life years) in young people aged between 10 and 24 years, representing 15·5% of the total DALY burden for all age groups [3]. Often referred to as “young people,” this group undergoes rapid changes in biological, psychological, and social factors, making it an important period of transition in their lives. From a health system perspective, it is an important phase as investments in health of young people offer lasting benefits for the young people not only through their lifetime, but also pass on to the next generation. Good health outcomes at this age also prepare a healthy workforce much needed for economic growth in the climate of global financial uncertainties. For years, this distinct and demanding phase has been unrecognized by the health systems in many parts of the world, where there are different specialities for childhood and adult health services, but no dedicated services for young people [4]. However, this is changing and the need for services specifically tailored for this phase of transition in life is being increasingly recognized [5–9]. Against a backdrop of increasing focus on unique health issues of young people, the growing complexity of their needs, and ever-increasing financial pressures on health systems, there is a need to deliver better care in a more effective, feasible, and acceptable way [10, 11].

Integrated care is one of the approaches suggested to achieve better patient care and greater efficiency from health delivery systems [12–17]. By linking providers to integrated networks, integrated care offers promise to improve the efficiency of systems by optimizing resource allocation. World Health Organization (WHO) defines integrated care as “Integrated care is a concept bringing together inputs, delivery, management and organization of services related to diagnosis, treatment, care, rehabilitation and health promotion” [18]. It covers a complex field, with many alternate terms like collaborative care, coordinated care, transmural care, seamless care, or comprehensive care.

Recent reports on health systems have focused attention on the integration of health care across traditional sectorial boundaries and on the increased provision of healthcare in out-of-hospital spaces [19, 20]. Out-of-hospital provision may include services with other pediatric services or more youth-specific services provided in a range of settings including schools. Although the impact of integrated services has been examined in the general population [21, 22], the implications of this for young people have not been systematically examined, and to date, there is no published systematic review which evaluates young people-specific, integrated out-of-hospital services. To fill this gap, we propose to undertake this systematic review with the objective to assess the effectiveness, feasibility, and acceptability of young people-specific integrated out-of-hospital services.

### Review question

What are the feasibility, acceptability, and effectiveness of out-of-hospital integrated services specifically for young people?

## Methods/design

The review protocol is reported according to the Preferred Reporting Items for Systematic Review and Meta-Analysis Protocols (PRISMA-P) guidance [23]. The completed checklist can be found in Additional file 1. A pre-defined set of eligibility criteria will be used to screen studies for inclusion in the review (see Table 1).

**Table 1:** Inclusion and exclusion criteria for the selection of studies for the review

Characteristic	Inclusion criteria	Exclusion criteria
Population	Young people (defined as 10–24 years)^a^ Human populations	Population other than young people (10–24 years) Animal studies
Health services	1. Health services outside of acute hospital settings, i.e., in the out-of-hospital space services provided in a range of settings including schools 2. Integrated, i.e., include an element of coordination between/across different health and social care sectors	Hospital-based health services, services other than integrated health services
Outcomes	Studies describing outcomes related to effectiveness, feasibility, and acceptability of health services. Some examples are mentioned below. Effectiveness: survival rate, rate of emergency department visit, rate of readmission, quality of life Acceptability: participation rate, satisfaction rate, compliance rate Feasibility: logistic challenges, coverage, staffing, time constraints	Any other outcomes
Study design	Experimental (randomized controlled trials, quasi-randomized controlled trials, non-randomized clinical trials) Quasi-experimental (interrupted time series, controlled before-after studies) and observational (cohort, case-control, cross-sectional, case series) Qualitative studies	Reviews, meta-analyses, and overviews^a^ Opinion pieces (i.e., commentaries, editorials, letters to editor)
Publication date	All	No exclusion on publication date
Language	Articles published in the English language	Articles published in any language other than English

^a^We will mine systematic reviews on topics relevant to our review, to identify primary studies for inclusion

^a^Source: Adolescence and youth demographics: A brief overview https://www.unfpa.org/sites/default/files/resource-pdf/One%20pager%20on%20youth%20demographics%20GF.pdf

### Search strategy

We designed our strategy following a series of initial scoping searches and with inputs from experts. We included keywords and controlled vocabulary terms for various databases to capture all words describing the key concepts: young people and integrated out-of-hospital health services. We will search the following electronic databases: MEDLINE, Excerpta Medica database (EMBASE), Cumulative Index to Nursing and Allied Health Literature (CINAHL) Plus, and Cochrane Controlled Trials Register (CENTRAL). Search strategy for all included databases is available in Additional file 2. We shall also search systematic reviews of relevant topics, along with citations and reference lists of included articles to identify eligible studies.

### Study selection process

The titles and abstracts of studies retrieved by search will be stored and managed in EPPI-Reviewer 4 software. After de-duplication, titles and abstracts will be screened independently by two reviewers (AP and AA) to identify studies eligible for full-text review based on predefined eligibility criteria. Any discrepancy in the classification of articles will be discussed and resolved by mutual consensus. Full-text articles for eligible studies will be obtained. Full-text articles will be assessed independently by two reviewers (AP and AA) against the study inclusion criteria. For the studies not satisfying inclusion criteria, an exclusion justification code will be allotted. Any disagreements regarding the study inclusion will be solved by mutual discussion, or by contacting a third reviewer (RV) if necessary. In case of insufficient details to determine eligibility, corresponding authors will be contacted for further details. There will be no blinding of review authors to author name, institution, or journal title.

### Data extraction and quality assessment

Data will be extracted for included studies using piloted data extraction forms. The following data will be extracted from the included studies: author(s), year of publication, country, objectives, study design, population, health services setting, health services objectives, follow-up data collection points, and effect measures regarding feasibility, acceptability, and effectiveness. If the data reported in published reports is insufficient, authors will be contacted by e-mail to obtain further details. Three such attempts will be made. If there is no response from the authors, then the study will be deemed ineligible.

Two reviewers (AP and AA) will extract the data and conduct quality assessment. Data extraction forms will be piloted on a sample of included studies (25%) to ensure that all the relevant information is captured and there is consistency in data extraction. The consistency of data extracted will be assessed to ensure > 95% agreement. The exporting, analysis, and outputs of the data extraction forms will also be pilot tested, on 25% subsample of included studies. Quality assessment of included studies will be done using Cochrane Effective Practice and Organisation of Care (EPOC) checklist for randomized controlled trials, non-randomized studies, controlled before and after studies and interrupted time series [24]. Quality assessment will be done by both reviewers together with discussion and any disagreement will be resolved by mutual consensus or discussion with a third reviewer (RV). Qualitative studies will be appraised using the Critical Appraisal Skills Programme (CASP) checklist [25]. The quality assessment would aid in the interpretation of results, but will not be used to determine inclusion.

### Data synthesis and analysis

Data reporting on indicators of feasibility, acceptability, and effectiveness will be summarized narratively, and an overview of young people-centered, out-of-hospital integrated health services will be provided with details about the setting, components of health services, service providers and outcomes. Wherever possible, we will synthesize quantitative data using pooled estimates and forest plots. For outcomes relating to utilization of health services, like participation rate, cure rate, and OPD attendance, quantitative data will be pooled and analyzed together, if data is deemed eligible for such quantitative synthesis. Studies reporting similar outcomes will be grouped with each other, e.g., studies describing waiting times and cure rates. Further, data from studies reporting different outcome measures of effectiveness, feasibility, and acceptability will be combined. The youth-specific out-of-hospital integrated health services may vary according to target age groups, type of health conditions, and by settings in which they are delivered. If sufficient data is available for such comparisons, subgroup analysis will be conducted.

## Discussion

To our knowledge, this will be the first review to synthesize evidence on the evaluation of young people-specific out-of-hospital integrated health services. The provision of youth-specific out-of-hospital integrated health services can improve service delivery and access, and the evidence synthesis of evaluation of such services can help identify models or key components of models which have been most effective, acceptable, and feasible. The findings of this review can help identify the areas where interventions for youth-specific integrated care are most effective, in terms of the settings, service delivery, and care models. Information about the characteristics of interventions will help policy makers, researchers, and health professionals in the design and selection of models of integrated health care for young people. Differences in the service patterns may guide on how to adapt in various “out-of-hospital” settings. We do recognize that the concept of “integrated” care is complex, and there is a lack of uniformity in its definition and interpretation. Also, the models of integrated care in itself are likely to be highly complex with multiple elements making it difficult to classify interventions. These are likely to be few of the challenges while interpreting the evidence on integrated services and summarizing it. Our review will also identify any gaps in the existing evidence to provide direction for future research.

### Strength of cumulative evidence

All relevant studies will be included in our review, regardless of the results of the quality assessment. However, quality assessment scores will be used to interpret the results, recognizing the limitations of the quality of primary studies.

## Additional files

Additional file 1: Completed PRISMA-P Checklist. (DOCX 30 kb)

Additional file 2: Electronic Search Strategy for various databases searched. (DOCX 18 kb)

## Abbreviations

CENTRAL: Cochrane Central Register of Controlled Trials
CINAHL: Cumulative Index to Nursing and Allied Health Literature
EMBASE: Excerpta Medica database
EPHPP: Effective Public Health Practice Project
PRISMA-P: Preferred Reporting Items for Systematic Review and Meta-Analysis Protocols
WHO: World Health Organization
