# Supplementary material for: Feasibility, acceptability, and effectiveness of young people-specific, integrated out-of-hospital services: a protocol for a systematic review
Source: Syst Rev. 2019 Mar 28;8:77. doi: 10.1186/s13643-019-0993-9 (PMC6437859; doi:10.1186/s13643-019-0993-9)
Supplement: Supplementary file 2 — Electronic Search Strategy for various databases searched. (DOCX 18 kb) [file 13643_2019_993_MOESM2_ESM.docx]

**Additional File 2: Electronic Search Strategy for databases:**

1. MEDLINE

|  | **MEDLINE Via PubMed** |
| --- | --- |
|  | "Delivery of Health Care, Integrated"[Mesh] OR “delivery of care” OR  "delivery of healthcare" OR "Comprehensive Health Care"[Mesh] OR "comprehensive healthcare" OR "comprehensive care" OR "comprehensive health" OR "Continuity of Patient Care"[Mesh]OR "continuity of patient care" OR "continuity of care" OR "continuity of health" OR "continuity of healthcare" OR “cross sectoral care” OR "intersectoral care” OR “integrated care*”OR “integrated health” OR “integration of care” OR multi team OR “integrated service network” “multiagency care” OR multiteam OR “multi care” OR “integrated services” OR “intersectoral care” OR multicare OR “multi-agency care” “multi clinic” OR multiclinic OR “multi service” OR multiservice OR “multi program” OR multiprogram OR “multi programme” OR “multi delivery” OR multidelivery OR “multi management OR “outreach services” |
|  | “coordinated care” OR “co-ordinated care” OR coordinat* care OR "coordination of care" OR “care co-ordination” OR coordinat* services OR "coordination of services" OR coordinat* programmes OR coordinat* programs OR “coordination of programmes” OR “coordination of programs” OR “coordination of service delivery” OR coordinat* services OR “coordination of services” OR coordinat* service delivery OR coordinat* delivery OR “chains of care” OR “collaborative care” OR “care coordination” OR “care transition” OR “cooperative care” |
|  | “horizontal service delivery” OR “horizontal delivery” OR “horizontal care” OR “horizontal integration” OR “horizontal services” OR “horizontal management” OR “horizontal programmes” OR “horizontal programs” OR “vertical services” OR “vertical programmes” OR “vertical programs” OR “vertical care” OR “vertical service delivery” OR “vertical services” OR “vertical management” OR “vertical integration” OR “clinical integration “ OR “financial integration” OR “functional integration” |
|  | 1 OR 2 OR 3 |
|  | Child/ |
|  | Adolescent/ |
|  | Minors/ |
|  | Young adult/ |
|  | exp PEDIATRICS/ or exp PUBERTY/ |
|  | (adolescen* or teen* or youth* or young or juvenil* or minors* or highschool* OR boy OR boys OR girl* OR kid OR kids OR child OR child* OR children* OR schoolchild*).ti,ab,jw,nw. |
|  | (paediatric*OR pediatric).ti,ab,jw,nw |
| 12 | 5 OR 6 OR 7 OR 8 OR 9 OR 10 OR 11 |
| 13 | 4 AND 12 |

1. EMBASE:

| **II.** | **EMBASE via Ovid** |
| --- | --- |
|  | ("Delivery of Health Care, Integrated"[Mesh] OR “delivery of care” OR  "delivery of healthcare" OR "Comprehensive Health Care"[Mesh] OR "comprehensive healthcare" OR "comprehensive care" OR "comprehensive health" OR "Continuity of Patient Care"[Mesh]OR "continuity of patient care" OR "continuity of care" OR "continuity of health" OR "continuity of healthcare" OR “cross sectoral care” OR "intersectoral care” OR “integrated care*”OR “integrated health” OR “integration of care” OR multi team OR “integrated service network” “multiagency care” OR multiteam OR “multi care” OR “integrated services” OR “intersectoral care” OR multicare OR “multi-agency care” “multi clinic” OR multiclinic OR “multi service” OR multiservice OR “multi program” OR multiprogram OR “multi programme” OR “multi delivery” OR multidelivery OR “multi management OR “outreach services”).ti,ab,jx |
|  | (“coordinated care” OR “co-ordinated care” OR coordinat* care OR "coordination of care" OR “care co-ordination” OR coordinat* services OR "coordination of services" OR coordinat* programmes OR coordinat* programs OR “coordination of programmes” OR “coordination of programs” OR “coordination of service delivery” OR coordinat* services OR “coordination of services” OR coordinat* service delivery OR coordinat* delivery OR “chains of care” OR “collaborative care” OR “care coordination” OR “care transition” OR “cooperative care”).ti,ab,jx |
|  | (“horizontal service delivery” OR “horizontal delivery” OR “horizontal care” OR “horizontal integration” OR “horizontal services” OR “horizontal management” OR “horizontal programmes” OR “horizontal programs” OR “vertical services” OR “vertical programmes” OR “vertical programs” OR “vertical care” OR “vertical service delivery” OR “vertical services” OR “vertical management” OR “vertical integration” OR “clinical integration “ OR “financial integration” OR “functional integration”).ti,ab,jx |
|  | 1 OR 2 OR 3 |
|  | exp Child/ |
|  | exp Adolescence/ |
|  | exp Adolescent/ |
|  | (child* OR children* OR adolescen* OR teen* OR youth* OR young OR juvenile* OR minors OR highschool* OR schoolchild* OR boy OR boys OR girl* OR kid OR kids).ti,ab,jx |
| 9 | (p?ediatric* OR pubert* OR pubescen*).ti,ab,jx |
| 10 | exp PEDIATRICS/ or exp PUBERTY/ |
| 11 | 5 OR 6 OR 7 OR 8 OR 9 OR 10 |
| 12 | 4 AND 11 |

1. **CINAHL PLUS Via EBSCO**

|  | **CINAHL PLUS Via EBSCO** |
| --- | --- |
| S6 | S1 AND S5 |
| S5 | S2 OR S3 OR S4 |
| S4 | MH integrated healthcare delivery system OR MH integrated health care model OR MH integrated health service OR MH integrated healthcare system OR MH integrated health systems OR MH integrated health OR MH healthcare delivery system OR MH comprehensive care OR MH integrated care model OR AB integrated care OR AB comprehensive healthcare OR AB comprehensive health OR MH continuity of patient care OR AB cross sectoral care OR AB intersectoral care OR AB multi care OR MH multiagency OR AB multi-agency care OR AB multi clinic OR AB multiservice OR AB multiservice OR AB multi program OR AB multiprogram OR AB multi programme OR AB multi delivery OR AB multidelivery OR AB multi management OR AB outreach services |
| S3 | MH coordinated care OR AB co-ordinated care OR AB coordinat* care OR AB coordination of care OR AB care co-ordination OR AB coordinat* services OR AB coordination of services OR AB coordinat* programmes OR AB coordinat* programs OR AB coordination of programmes OR AB coordination of programs OR AB coordination of service delivery OR AB coordinat* services OR AB coordination of services OR AB coordinat* service delivery OR AB coordinat* delivery OR AB chains of care OR AB collaborative care OR MH care coordination OR MH care transitions OR AB cooperative care |
| S2 | AB horizontal service delivery OR AB horizontal delivery OR AB horizontal care OR AB horizontal integration OR AB horizontal services OR AB horizontal management OR AB horizontal programmes OR AB horizontal programs OR AB vertical services OR AB vertical programmes OR AB vertical programs OR AB vertical care OR AB vertical service delivery OR AB vertical services OR AB vertical management OR AB vertical integration OR MH clinical integration OR AB financial integration OR MH functional integration |
| S1 | MH paediatrics or children or child or young person OR MH paediatric or pediatric or children or child OR MH adolescents or young people or teen or young adults OR MH puberty OR MH minors OR MH girls OR MH boys OR MH high school students or teenagers or secondary schools or adolescents OR MH juvenile OR AB adolesc* |

1. Cochrane CENTRAL:

| **IV. Cochrane Central Search Strategy** |
| --- |
| #1 MeSH descriptor: [Delivery of Health Care, Integrated] this term only  #2 MeSH descriptor: [Continuity of Patient Care] this term only  #3 MeSH descriptor: [Comprehensive Health Care] this term only  #4 (delivery of care OR delivery of healthcare OR comprehensive healthcare OR comprehensive care OR comprehensive health OR continuity of patient care OR continuity of care OR continuity of health OR continuity of healthcare OR cross sectoral care OR intersectoral care OR integrated care OR integrated health OR integration of care OR multi team OR integrated service network OR multiagency care OR multiteam OR multi care OR integrated services OR intersectoral care OR multicare OR multi-agency care OR multi clinic OR multiclinic OR multi service OR multiservice OR multi program OR multiprogram OR multi programme OR multi delivery OR multidelivery OR multi management OR outreach services):ti,ab,kw  #5 (coordinated care OR co-ordinated care OR coordinat* care OR coordination of care OR care co-ordination OR coordinat* services OR coordination of services OR coordinat* programmes OR coordinat* programs OR coordination of programmes OR coordination of programs OR coordination of service delivery OR coordinat* services OR coordination of services OR coordinat* service delivery OR coordinat* delivery OR chains of care OR collaborative care OR care coordination OR care transition OR cooperative care):ti,ab,kw  #6 (horizontal service delivery OR horizontal delivery OR horizontal care OR horizontal integration OR horizontal services OR horizontal management OR horizontal programmes OR horizontal programs OR vertical services OR vertical programmes OR vertical programs OR vertical care OR vertical service delivery OR vertical services OR vertical management OR vertical integration OR clinical integration OR financial integration OR functional integration).ti,ab,kw  #7 #1 OR #2 OR #3 OR #4 OR #5 OR #6  #8 MeSH descriptor: [Child] this term only  #9 MeSH descriptor: [Adolescent] this term only  #10 MeSH descriptor: [Young adult] this term only  #11 (adolescen* or teen* or youth* or young or juvenil* or minors* or highschool* OR boy OR boys OR girl* OR kid OR kids OR child OR child* OR children* OR schoolchild*).ti,ab,kw  #12 #9 OR #10 OR #11  #13 #7 AND #12 |
|  |
